# Paralysis of Arm Resulting from a Carious Tooth

**Published:** 1865-11

**Authors:** 


Paralysis of Arm resulting from a Carious Tooth.—At a meeting of the Medical Society of the county of Kings, Dr. Whitney related a case of partial paralysis of the arm, consequent upon caries and exposure of the nerve of the lower dens sapientia of the right side, which Dr. Peters, the Secretary of the Society reports in the *Buffalo Medical and Surgical Journal.*

The patient was a thin, spare woman, 40 years of age, nervous temperament, had had but very little pain in the tooth, but for several weeks or months considerable pain in the right side of the neck, extending to the shoulder and arm, with rigidity of the muscles, and, at times, immobility of the arm. On raising the hand to the face to locate the pain it fell to her side. On coming in contact with the nerve in probing the tooth, the effect was more manifest in the arm than in the tooth, by painful twitching of the muscles, with inability to raise it, so much so that she took hold of it with the other hand. The usual mode of devitalizing and removing the pulp and filling the cavity entirely removed the symptoms in the arm.— *Med. and Surgical Reporter.*

